# In This Issue/Abstract Thinking: Treatment Response in Psychiatry

**DOI:** 10.1016/j.jaac.2012.03.014

**Published:** 2012-06

**Authors:** Argyris Stringaris

**Affiliations:** Institute of Psychiatry, King's College London, London, UK

## In This Issue

It is common for medical categories to be heterogeneous. A classic example is leukemia: there are different kinds of leukemias, each with a different etiology and a different clinical course. Disruptive behavior disorders seem an analogous example from child and adolescent psychiatry.

Previous work has suggested that disruptive behaviors are made up of distinguishable dimensions of feelings and behaviors (such as callous and unemotional traits or irritable mood). In this issue of the *Journal*, Wakschlag and colleagues (p. 593) are taking this approach further in two important ways. First, they are examining preschoolers, an underinvestigated age group. Second, the investigators have a clear theory that they set out to test by examining the fit of different models. They find that their approach of separating out the heterogeneity within disruptive behavior disorders is superior to the three competing models (including the conduct disorder/oppositional defiance disorder distinction present in *DSM-IV*). In particular, the investigators provide evidence that disruptive behavior disorders in preschool children may be best seen as comprising four distinguishable dimensions of feelings and behaviors: temper loss, noncompliance, aggression, and low concern for others. The investigators examine the reliability and validity of their distinctions using two different samples (one clinic based and the other community based), which helps solidify their findings. One of the important next steps, as the investigators also remark, will be ways that could help exploit these insights for clinical practice.

Another source of heterogeneity can be the way in which psychiatric problems are measured. This is a methodologic source of heterogeneity that can have profoundly negative implications—subjects may have the same diagnostic labels but be dissimilar in their symptom profiles and, thus, study findings may be inconsistent. The study by Galanter and colleagues (p. 605) in this issue of the *Journal* attempts to tackle this major question for bipolar disorder. As every child psychiatrist knows, some of the hardest problems in assessing bipolar disorder include how to establish whether a child's irritability is episodic rather than a trait, when exactly an episode started, how long such an episode lasted, how exactly to establish episodicity (a change in baseline), and which of the potentially manic symptoms (e.g., grandiosity or disinhibition) were present during this very episode of a change in mood. The review by Galanter and colleagues shows that there are ambiguities in *DSM-IV-TR* mania criteria and that these are reflected in the different ways in which psychiatric instruments assess these problems. The investigators conclude with a list of clear recommendations on how to best assess bipolar disorder for future instruments and the *DSM-5*. The investigators also discuss issues that relate to the minimum training required for people to use such instruments and the best ways to reach consensus about these matters in the field.

Differences in pathophysiologic processes can underlie heterogeneity. Strawn and colleagues (p. 642) in this issue of the *Journal* use magnetic resonance spectroscopy to show that brain glutamate levels may predict whether divalproex sodium will be successful for the treatment of mania in adolescents. The investigators found that glutamate levels in patients were no different from those in healthy volunteers at baseline. However, patients with lower glutamate levels in the left ventrolateral prefrontal cortex, an area implicated in mood regulation, were more likely to remit after treatment with divalproex. Moreover, improvements on the in the Youth Mania Rating Scale correlated positively with changes in glutamate concentrations in remitting patients. The findings are important for two reasons. First, they demonstrate a potential source of person-specific heterogeneity in treatment outcome—brain glutamate levels. Second, they remind us that measuring such markers is feasible in vivo using magnetic resonance spectroscopy. The clinical utility of such a measurement and the extent to which baseline glutamate levels may be a worthwhile target for intervention remain to be examined.

## Abstract Thinking

### Treatment Response in Psychiatry

Treatment response can vary within individuals who have the same diagnostic label because of heterogeneity. Severity is yet another source of heterogeneity and it is often thought to influence treatment response in psychiatric disorders such as depression. Gibbons and colleagues^1^ used person-level longitudinal data from the first 6 weeks of treatment of antidepressant trials for adolescents, adults, and geriatric patients. They reported several important findings. Firstly, antidepressants were effective for the treatment of depression in any age group. Secondly, the response and remission rates were largest for adolescents. For example, 46.6% had a remission on fluoxetine versus 16.5% of adolescents treated with placebo, amounting to a number needed to treat of 3.33. Thirdly, there was no evidence that severity had an influence on treatment outcome: patients with low or high severity responded to a similar extent.

These findings lead to another, more general, question: How well do treatments work for psychiatric patients? Questions about the heterogeneity of psychiatric phenotypes and the relatively sparse knowledge about the etiology of psychiatric disorders typically come with concerns about the efficacy of the treatments psychiatrists offer. Thus far, little has been done to address this issue in an empirical manner. In a recent article, Leucht and colleagues^2^ reformulated the question as follows: How well do psychiatric treatments work compared with treatments in other medical disciplines? To answer this question, the investigators conducted a review of 94 meta-analytic studies of drugs compared with placebo for common medical and psychiatric disorders. There were two striking findings. The first was that, overall, psychiatric drugs were no less efficacious than other drugs. An example that puts this in perspective is that the acute treatment of schizophrenia with antipsychotics led to a standardized mean difference of around 0.5 compared with 0.11 for the thrombolysis of acute stroke and 0.56 for the treatment of asthma with corticosteroids. The second finding is of particular interest to child psychiatrists: the treatment of attention-deficit/hyperactivity disorder was the most efficacious overall, with a standardized mean difference of 0.78.

These are encouraging findings for the field of child and adolescent psychiatry and for what it has to offer to young patients. However, there is still a lot of work ahead. A particular challenge is using evidence-based treatments for as many patients as possible. In other areas of medicine—such as cancer treatment—this is achieved by including most patients in ongoing large-scale trials. Doing so not only advances pharmacotherapy (this is the way to rigorously test medications) but also improves patients' outcomes because it is known that those taking part in trials do better than those who do not participate in trials.^3^

## References

[1] Gibbons RD, Hur K, Brown CH, Davis JM, Mann JJ. Benefits from antidepressants: synthesis of 6-week patient-level outcomes from double-blind placebo-controlled randomized trials of fluoxetine and venlafaxine [published online ahead of print March 5, 2012]. Arch Gen Psychiatry. doi:10.1001/archgenpsychiatry.2011.2044 10.1001/archgenpsychiatry.2011.2044 PMC3371295 22393205

[2] Leucht S., Hierl S., Kissling W., Dold M., Davis J.M. (2012). Putting the efficacy of psychiatric and general medicine medication into perspective: review of meta-analyses. Br J Psychiatry.

[3] Stiller C.A., Draper G.J. (1989). Treatment centre size, entry to trials, and survival in acute lymphoblastic leukaemia. Arch Dis Child.

